# New products

**DOI:** 10.1155/S1463924689000313

**Published:** 1989


Journal of Automatic Chemistry Vol. 11, No. 3 (May–June 1989), p. 137–147

### New products

### New mini-Ebdon nebuliser - A direct replacement for a Meinhardt but with added attractions

Following the success of the PSA 60.006 Ebdon High Solids Nebuliser in relationship to slurry atomisation for ICP, PS Analytical, in co-operation with Plymouth Polytechnic, has recently developed a mini-Ebdon version of this nebuliser. Designed to fit directly into the Meinhardt nebuliser end-cap as a straight replacement and constructed from Kel-F, the nebuliser both performs well and offers significant advantages over the Meinhardt and crossflow nebulisers.

Data obtained from a sequential plasma emission spectrometer using a Gilson Minipuls (Luton, Bedfordshire) peristaltic pump to aspirate the sample into a Fassel-type torch is presented in table 1. The work was carried out with a reduced-size spray chamber coupled to a flow injection valve. Detection limits were obtained in the standard continuous mode of operations, however, 10 μl samples of 100 ppb concentration can be aspirated easily. The mini-Ebdon, like the standard version, is inert, is highly resistant to blockages, and provides similar detection level capabilities to the Meinhardt nebuliser.

For a limited period these nebulisers will be offered at an introductory price of £210·00 delivered to the UK.

*Table1.*

| Element | Limit of detection in ppb (based on 3 σ) |
|---|---|
| Cu | 5 |
| Pb | 148 |
| Mn | 4 |
| Ca | 0·29 |
| Mg | 0·040 |

Comparison of data with Meinhardt using similar plasma conditions give a detection level for Mg of 0·086 ppt.

*For information on this offer and further information on this new product, contact PS Analytical at their new address: Arthur House, B4 Chaucer Business Park, Watery Lane, Kemsing, Sevenoaks, Kent TN15 6QY. Tel.: 0732 63416; Telex: 957645 PSA G; Fax: 0732 61340.*

### Fluorescence detector from Philips Analytical

The PU4027 fully programmable fluorescence detector from Philips achieves high sensitivity and selectivity through the use of high energy efficient optics. Designed for the analysis of native and derivatised fluorescent compounds, the PU4027, in combination with solvent delivery systems such as the PU4100, provides the ultimate in LC trace analysis.

Microprocessor-controlled operation gives greater versatility, excellent reliability and ease of use, while operation is further enhanced by an LCD diagnostic multi-display. True dual monochromator operation, together with scanning of fluorescence spectra, gives unlimited flexibility when determining optimum detection conditions for multicomponent analyses.

A major feature of the PU4027 is the ability to change wavelengths automatically during the chromatographic run. The instrument can be programmed to automatically change excitation and emission wavelengths prior to peaks appearing. Under these conditions, components can be detected at maximum sensitivity.

*For further information, contact: Philips Scientific, York Street, Cambridge CB1 2PX, UK. Tel.: 0223 358866.*

### Polyscan 61E spectrometer from Thermo Jarrell Ash

Thermo Jarrell Ash have announced the PolyScan 61E spectrometer, a simultaneous/sequential spectrometer for elemental analysis of water, oil and other materials. The spectrometer consists of a 3/4 meter polychromator for high sample throughput and a 3/4 meter scanning monochromator for the characterization and analysis of samples for elements which have wavelengths that are not installed in the focal curve of the polychromator. The monochromator and polychromator share the same ICP source and sample introduction system and are controlled using our ThermoSPEC software. The entire system, including RF power, gas flows, and sample introduction system are controlled via an IBM Personal System/2 Model 50 microcomputer. The plasma torch can be operated with low power, low argon flow for routine samples, and high power, high argon flow for difficult samples.

Unattended operation from the autosampler provides all the quality control logic functions required by the EPA Contract Laboratory Program. Our ThermoSPEC software offers multi-level operation including menu-driven control for novices and occasional users, and a command mode for expert spectroscopists. Also included is an integrated word processor, database manager, spreadsheet, graphics and telecommunications package.

*For further information contact: Thermo Jarrell Ash Corp., 8E Forge Parkway, Franklin, MA 02038, USA. Tel.: 508 520 1880.*

### Hewlett-Packard introduces bench top atomic emission detector

Hewlett-Packard company has introduced the first totally automated multi-element atomic emission detector (AED) for gas chromatography. The HP 5921A is capable of selectively detecting any element (except helium) in gas chromatographic effluents.

The AED combines a new atomic emission source with a flat focal-plane

0142–0453/89 $3.00 © 1989 Taylor & Francis Ltd.

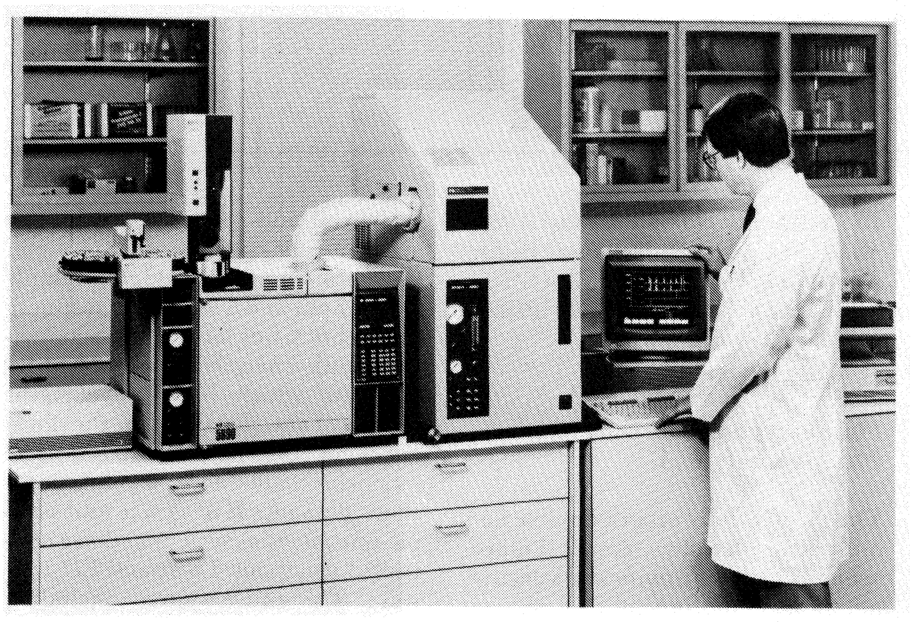

*The new HP 5921A atomic-emission detector from Hewlett-Packard Company with complete system components includes: power supply for autosampler, left, HP 5890A gas chromatograph, HP 5921 atomic-emission detector and the computer controller and software. (PRAN43808)*

spectrometer containing a movable photodiode array. Controlled by the HP ChemStation, the AED, autosampler and gas chromatograph provide total automation from element selection through report generation.

HP's new detector is the first of its kind capable of detecting oxygen, nitrogen, sulphur, carbon, silicon, hydrogen, chlorine, bromine and many other elements at picogramme levels. Applications include petroleum and petrochemical processing, environmental monitoring and chemical manufacturing. It is particularly useful as an aid in the identification of chemical compounds.

Product capabilities: detection limits from 0·5 pg/s for carbon to 100 pg/s for fluorine; selectivities of 10 000 for most elements, including oxygen, when compared to carbon; detection of up to four elements simultaneously from one injection; automatic sequences through any number of elements per sample by changing wavelength and reinjecting; on-line spectra to confirm the presence of a particular element; response factors for the same element in different compounds are constant to within 10%, regardless of the chemical nature of the compound being analysed; a compound's element ratio can be determined to typically better than 10% accuracy; column compatibility ranges from 100-μm ID fused silica capillary to ¼-inch packed columns; total automation of analysis and customised data presentation through macro-programming capabilities.

The AED consists of three major parts: the light source (microwave-induced plasma); the light separator (spectrometer); and the light sensor [photodiode array (PDA)]

The light source for the HP 5921A is an atmospheric pressure, microwave helium-induced plasma. This plasma is generated in a water-cooled discharge tube contained in a new cavity design called a re-entrant cavity, which is a wave-guide coupled to a magnetron supplying microwave power. This arrangement provides for a very stable atomic emission source and requires no tuning of microwaves.

The spectrometer is a flat focal-plane mount consisting of an autofocusing slit, a concave holographic grating and a movable PDA. The latter covers a portion of the spectrum (ranging between 10 and 30 nm, depending on wavelength) in the 160–800 nm region. Any group of elements that have emission lines within the span of the PDA can be detected simultaneously, up to a maximum of four elements. Every element on the periodic table has at least one atomic emission line in the spectral range of this design.

Using a PDA as the sensor gives real-time multipoint background correction to provide high selectivities. Since the PDA output can be saved during a run, these on-line spectra can be used to confirm the presence of a given element in the gas-chromatographic peaks.

The HP 5921A complements mass spectrometry and infrared detection by allowing the user to determine elemental composition, which will be complementary to IR and mass-spectral techniques.

The system, which is priced below $100 000, includes the HP 5921A atomic emission detector, HP 5890A gas chromatograph, computer controller with HP 35920A GC/AED software, HP 7673A automatic injector, and HP ThinkJet printer/plotter. Estimated delivery is 16 weeks ARO.

*For further information, contact: Hewlett-Packard, 3000 Hanover Street, Palo Alto, CA 94304, USA. Tel.: 415 857 1501.*

### Preparative HPLC software

The Varex Corporation has just released the high resolution EGA graphics version of their VERSAPrep Controller/Data Acquisition Software. This software additionally has new features such as the display of a second detector signal and the ability to loop and link stored methods.

This software now comes standard with all VERSAPrep HPLC Systems and is compatible with current installations of the VERSAPrep.

The Varex VERSAPrep has been designed for high-performance preparative separations using high efficiency packing materials. Two features not found in any other available instrument are the pre-column switching valve with backflush and the multi-column switching valve. These options allow for unique and versatile approaches to solving preparative purification problems.

A 15 min video tape showing the VERSAPrep using column switching techniques is also now available.

*For further information, contact: Varex Corp., 12221 Parklawn Drive, Rockville, MD 20852, USA. Tel.: 301 984 7760.*

### Beckman software for automatic on-line dissolution analysis

The new Beckman Dissolution 6/12 Soft-Pac software module for use with the Beckman range of DU spectrophotometers provides convenient automatic on-line dissolution analysis from one or two baths. The software program integrates system operation and control, sample delivery, data acquisition and hard copy reporting. Analytical wavelength can be selected for productive analysis extending up to 166·6 h determined by the time interval and number of baths. Using one or two wavelengths, the Soft-Pac module also allows for automatic background correction of raised or sloping baselines caused, for example, by sample turbidity. Volume corrections are not required.

Data can be customised by the user to specific needs without complex programming steps. Dissolution percentage is calculated from a standard, or from a calibration curve generated by either linear regression or quadratic curve fitting, depending on the absorption characteristics of the compound under analysis.

Reports include raw absorbance data, reduced tabulated data and graphic plots of the dissolution profile. Test results include correction for reference standard weight, the mean and relative standard deviation, and minimum and maximum values. An optional RS232C output provides raw data to an external computer or data logger.

*For further information contact: Beckman, Progress Road, Sands Industrial Estate, High Wycombe, Bucks, UK. Tel.: 0494 441181.*

### Software package for determination of tristimulus values

An integrated software package from Beckman – the DU Data Leader together with Lotus 1-2-3 spreadsheet software – is designed for use in the determination of tristimulus values in the widely adopted CIE (Commission International de l'Eclairage) system.

Beckman's Data Leader is a powerful, fully integrated software package which allows data capture from Beckman spectrophotometers and subsequent mathematical and graphical manipulation (in full colour on an IBM personal computer), thereby freeing the spectrophotometer for its primary task of data measurement. Raw data files as well as manipulated files can be permanently stored on disk.

*For further information, contact: Beckman, Progress Road, Sands Industrial Estate, High Wycombe, Bucks, UK. Tel.: 0459 441181.*

### Communications software for Beckman's spectrophotometers

Beckman have recently added DU Data Comm, a communications package for the company's DU-60 Series spectrophotometers, to their extensive range of software options.

DU Data Comm is designed for use with an IBM PC/XT/AT or PS/2 Series personal computer, allowing the user to send programs stored on disk or in the Beckman Data Transporter to a DU-60 or DU-50 Series spectrophotometer. Programs can also be sent from the spectrophotometer to the personal computer or Data Transporter.

This capability allows mass storage of spectrophotometer step programs and provides an easier method of writing and editing such programs.

The seven functions available are shown on the main option screen and, by making a choice of a function letter, the user can receive a program from or send a program to the instrument, accept a program from the optional Data Transporter, dump a program to the transporter, get the program directory from the instrument, list a program on the printer, or change set-up parameters. Help messages can be obtained at any time during use at the touch of a key.

*For further information, contact: Beckman, Progress Road, Sands Industrial Estate, High Wycombe, Bucks, UK. Tel.: 0494 441181.*

### HP software for UV/visible spectroscopy

Hewlett-Packard Company have announced new MS-DOS software for the HP 8452A UV/visible spectrophotometer and the HP Vectra personal computer.

A combination of diode-array spectroscopy with applications software make it possible to offer a single package suitable for both laboratories requiring straightforward, reliable, high-productivity, routine analyses and laboratories involved in method development and research.

The new software contains options for general scanning, quantitation and kinetics applications. The package uses easy to follow menus and offers improved graphics features and better data-handling capabilities.

The general scanning software can display a complete UV/visible spectrum in seconds. With only a few keystrokes, spectra can be displayed, overlaid and mathematically manipulated displaying the resulting spectra. The quantitation software provides fast quantitative determinations at one or several wavelengths. Suspect results are automatically flagged, and linear and non-linear calibration graphs can be selected. The system can be automated to run calibration standards, unknowns and blanks unattended.

The kinetics software enables users to automatically acquire complete spectra at selected time intervals. Any wavelength can be selected from these data sets. Reaction rates and rate constants can be calculated for the time trace data. The resulting display includes a plot of the time trace data overlaid with the calculated curve fit.

Other programs can be started interactively from the UV/visible software environment. For example: complex calculations on spectral data and formatting can be done using spreadsheet programs, such as Lotus 1-2-3; files can be edited using several word-processing packages; spectra can be manipulated graphically using HP Charting Gallery or HP Drawing Gallery; files can be uploaded to mainframe computers using communication or networking packages. After using one of these packages, the display automatically returns to the UV/visible software without further programming.

*For further information, contact: Hewlett Packard, 3000 Hanover Street, Palo Alto, CA 94304, USA. Tel.: 415 857 1501.*

### pUMa CHEMMOD launched

U-Micro has announced the launch of an extremely powerful desk-top package.

The pUMa CHEMMOD is built around the Motorola 68020 and INMOS Transputer processors. The 68020 runs an industry standard multi-user, multi-tasking, operating system and controls peripherals, such as disks and printers. A T800 Transputer runs the main application code plus interfacing to the high performance graphics system built into the pUMa.

As well as providing a high level of performance the use of the Transputer gives the pUMa workstation an easily expandable computational power. The pUMa offers about 1·2 times the power of the VAX mini in its standard form but can be easily expanded to 100 times the power of the VAX. The Transputer makes this possible with its parallel processing architecture.

The CHEMMOD software package included in the pUMa CHEMMOD workstation provides comprehensive modelling facilities. It was first introduced in 1987 and has been sold successfully by U-Micro throughout the world.

A particular feature of the new pUMa CHEMMOD workstation is U-Micro's commitment to porting a wide range of other chemistry and modelling related packages. These include: MOPAC, a semi-empirical molecular orbital program which provides information not possible with molecular mechanics, such as charge distribution; Gaussian 88, a molecular orbital program; HASL, a QSAR program which extends the superpose facility of CHEMMOD to cover the Van der Waals volumes of the molecule, rather than just atomic positions; Predict, a protein secondary structure prediction program which, given the amino acid sequence, will predict the conformation of the individual amino acids; AMBER, molecular dynamics; 3DScan, a sophisticated program for searching the Leeds Birkbeck 3D protein structure database for features of interest; TP-MIN, a parallel version of the CHEMMOD minimiser for use with multiple transputers.

*For further information, contact: U-Microcomputers Ltd, 12 Chetham Court, Calver Road, Winwick Quay, Warrington, Cheshire WA2 8RF, UK. Tel.: 0925 54117.*

### Specialized thermal analysis software from Perkin-Elmer

Perkin-Elmer offer a range of specialized software programs for their DSC 7 Differential Scanning Calorimeter run by an Epson PC (TM) or IBM (R) PS-2. Purity software is used to determine the purity of most crystalline organic materials. The method is based on the van't Hoff law and does not require a pure standard of the material for calibration purposes. Results are quoted mole percent purity and can be plotted, together with the calorimetric data, on to an A4 plotter. Total analysis time is around 20 min, including sample preparation.

*For further information, contact: Perkin-Elmer Limited, Maxwell Road, Beaconsfield, Bucks HP9 1QA, UK. Tel.: 0494 676161.*

### Coulometric Karl Fischer Titrator for low-level moisture determinations

The Model 684 Karl Fischer titrator automates coulometric moisture measurements from 10 μg to 10 mg. The Model 684 provides results with 30 s at a maximum titration rate of 2 mg $min^{-1}$. It's features include elimination of standardization by generating it's own reagents, self-calibration (absolute measurements eliminate the need for calibration) and user-friendliness. An alpha-numeric readout displays results in percent or ppm water and cell conditions. There is no need for coding since titration procedure keys are clearly labelled on the front panel. The Model 684 can be connected to a computer or printer for data output.

The Karl Fischer Model 684 is ideal for the determination of water in solvents, pharmaceuticals, petrochemicals, chemicals and fats and oils.

*For further information, contact V. A. Howe and Co. Limited, 12–14 St. Ann's Crescent, London SW18 2LS, UK. Tel.: 01-874 04220.*

### Metrohm Karl Fischer titrator streamlines amperometric measurement

The Metrohm Model 658 Karl Fischer titrator streamlines amperometric measurement with operator prompts at every step of each procedure on a 16-digit alphanumeric display. It features a built-in standardization program in which results are calculated and printed in 1 min or less and automatically stored for future use, and measures solid, liquid or gaseous samples from 1 to 100 mg (0·001% to 100% $H_2O$).

The Model 658 is easy to operate; all procedures require just one keystroke, and a built-in printer documents all results. An electronic balance can be coupled to the Model 658 to further automate the system; automation can be completed by interfacing to a central or host computer.

*For further information, contact: V. A. Howe and Co. Limited, 12–14 St. Ann's Crescent, London SW18 2LS, UK. Tel.: 01 984 0422.*

### Oil monitoring simplified

Radiometer has introduced a dedicated oil analysis plug-in option package for its TitraLab automated laboratory system. With a TAN/TBN method module added, the TitraLab system can rapidly and accurately determine important oil data. For instance, strong acid number (SAN) and total acid number (TAN) or strong base number (SBN) and total base number (TBN) can now be determined in a single potentiometric titration. The results are given automatically in mgKOH/g.

*For further information, contact: Radiometer Ltd, Manor Royal, Crawley, West Sussex RH10 2PY, UK. Tel.: 0293 517599.*

### Laboratory robotic systems for automated titrations

Zymark Corporation has developed several laboratory robotic systems designed to automate general purpose and Karl Fischer titrations. Using PyTechnology hardware, these robotic systems handle the preparation of liquids, viscous liquids and solid samples. The samples are prepared by the robot, in a sequence that is specified by the operator, and placed directly into the titration cell. The robot starts the titrator and monitors all operations to ensure a proper titration. When the titration is complete, the results are sent back to the robot and full documentation is provided. As the robot has full control over the titrator, any problems that are encountered with the samples are reported to the operator.

Several key benefits of a fully automated titration system are: unattended operation frees up the lab personnel; unattended operation provides greater sample throughput; reproducible operations improves the accuracy and precision of the data; provides the full documentation of the results; and reduces the cost per sample, as more samples can be run with less operator intervention. Several commercially available titrators can be interfaced with the robotic system, offering a full line of titration capabilities.

*For further information, contact: Zymark Corporation, Zymark Center, Hopkinton, MA 01748, USA. Tel.: 508 435 9501.*

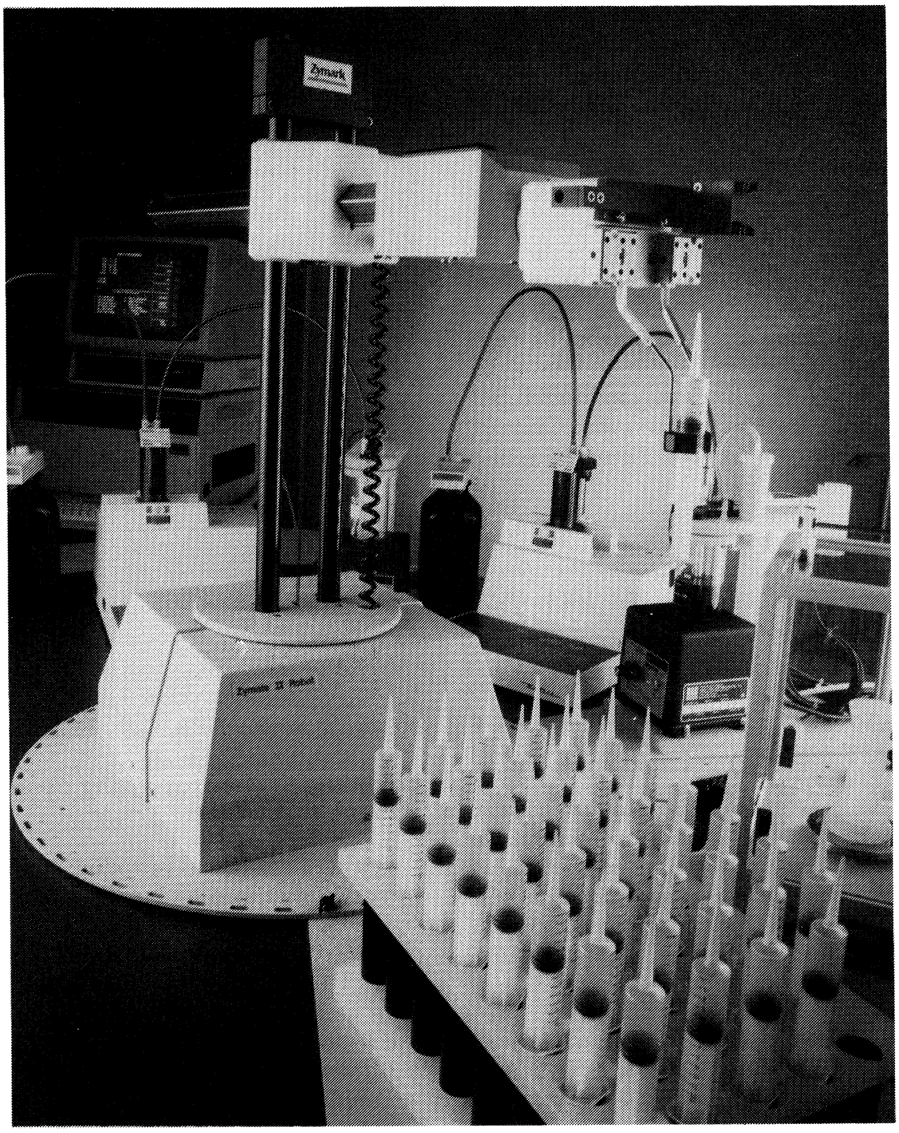

*Laboratory robotic systems for automatic titrations from Zymark.*

### Titration in Progress

A new 16-page colour brochure from Radiometer gives a full survey of all the titration systems offered by the company, and outlines selection criteria for the ideal titration system.

The brochure is available, free of charge, in either English or German. Data sheets for individual instruments are also available.

*For further information, contact: Radiometer Analytical A/S, 49 Krogshojvej, DK-2880 Bagsvaerd, Denmark. Tel.: 45 1 696311.*

### Accelerated surface area and porosimetry analyser

Micromeritics has introduced the ASAP 2000, an automated, single-port surface area analyser. The analyser can use a wide range of adsorbate gases, including nitrogen, krypton, oxygen, argon and carbon dioxide. A second analyser can be added to work simultaneously via the single control module. Up to 50 different report formats can be created and stored on-line.

Features include: multitasking software; 'smart dosing', a routine which calculates the rate of adsorption by the sample for each pressure point and then adjusts the volume of gas for the following dose; pre-defined analysis parameters (up to 50 run condition sets can be stored); and data archiving, where up to 6000 analysis data files can be stored on-line, up to 200 directories created and data can be converted into ASCII format.

Accurate results are obtained with the help of the saturation pressure tube, which is positioned alongside the analysis port so that measurements are made under exactly the same conditions as the analysis in progress. The use of patented isothermal jackets eliminates dead space error and allows analyses to run unattended for up to 60 hours. Independent vacuum systems isolate the sample prepar tion stations from the analysis port and eliminate the possibility of cross-contamination. Two distinct cold-traps prevent backstreaming of contaminating vapours.

*For further information, contact: Micromeritics, 1 Micromeritics Drive, Norcross, GA 30093-1877, USA. Tel.: 404 662 3660.*

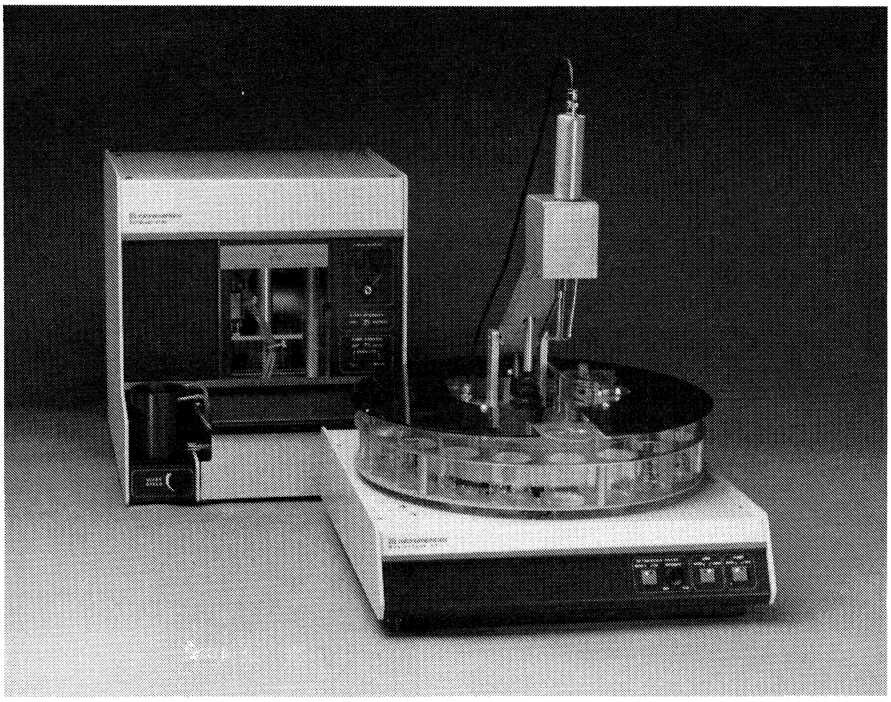

*Automatic sampler for particle size analysis (Micromeritics).*

### Automatic sampler for particle size analyser

Micromeritics has announced the MasterTech 51, an automatic sampler accessory for the SediGraph 5100 particle size analyser. The SediGraph 5100/MasterTech 51 system can analyse 1–18 samples automatically in sequence and completely unattended. When the SediGraph 5100 is ready for another sample it signals to the MasterTech 51. Mechanical and ultrasonic agitation built into the MasterTech 51 will redisperse the next sample to it's original level of dispersion. The MasterTech 51 then transfers the redispersed sample to the SediGraph 5100 for analysis and reporting. All of these operations, including backflush and rinse of the sample-transfer tubing and redispersion apparatus, are accomplished automatically. Control of all operations is handled by the SediGraph 5100 Controller/Data Management Module. Like hiring a Master Technician, the SediGraph 5100/MasterTech 51 system allows both unattended and after-hours operation. All analyses are performed exactly as specified without requiring the operator to be present. As with the SediGraph 5100, the MasterTech 51 will adapt to local voltages.

*For further information, contact: Micromeritics, 1 Micromeritics Drive, Norcross, GA 30093 1877, USA. Tel.: 404 662 3633.*

### Streamlined checkweighing operations

Sartorius Limited has introduced the Checkweigher into its family of precision balances and scales. The new product can be used with the Sartorius IB range of scales to provide a fast, easily identifiable method of check weighing. It uses a simple red/green/red display, which works in conjunction with pre-determined limits set into the scale, to indicate at a glance overweight, underweight or correct weight products.

To commence a typical checkweighing process, the operator first enters both the upper and lower acceptable weight limits into the keypad, tares the scale in order to take into account the weight of container and places the object to be weighed on to the pan. If the net weight displayed falls within the pre-programmed limits, the green indicator is illuminated on a separate display consol. If, however, the net weight is below the acceptable limit, a red light is displayed on the left-hand side of the unit and if heavier than required, a red light on the right-hand side of the unit shows. The programmed figures are retained by the Checkweigher until altered by the operator.

Together with the IB scales, the new Checkweigher has a weighing capacity to 31 kg to an accuracy of 0.1 g and has a multitude of applications. The light display console can actually be plugged into any Sartorius industrial scale, together with a 'plus performance' keypad to provide further checkweighing systems.

The Checkweigher can be used for mobile weighing using a rechargeable battery pack and the system can also be linked to a Sartorius Sartopac to provide an instant print-out of operations. This is particularly useful in average weight work for the purpose of statistics and for providing records for trading standards.

The system automatically performs a self-diagnostic check on power-up which ensures that consistent accuracy is maintained at all times. If, in the unlikely event of failure, a fault occurs, a code is displayed to indicate the area in which the problem has developed.

*For further information contact: Sartorius Limited, Longmead, Blenheim Road, Epsom, Surrey KT19 9QN, UK. Tel.: 03727 45811.*

### Electronic label printer for weighing data

Mettler have introduced an electronic label printer which can be connected to all the MultiRange precision scales for weights between 0·1 mg and 600 kg via the data interface option 089 of the MultiRange ID5 Control Terminal. The label printer uses EAN/UPC and all common industrial bar codes. The size and design of the printout can be determined by the user. Label lengths of 60 and 120 mm are possible with single tasking. There is a choice of 16 print sizes and character fonts.

*For further information, contact: Mettler Instruments AG, CH-8606 Greifensee, Switzerland. Tel.: 01 944 211.*

### Control terminal for weighing

The ID5 Control Terminal from Mettler can be used with all MultiRange weighing platforms. Three scales can be controlled via the single keypad terminal. The dynamic display acknowledges each function in clear text. There is an attachable alphanumeric strip printer to ensure reliable and certified result records of the weighing data.

*For further information, contact: Mettler Instruments AG, CH-8606 Greifensee, Switzerland. Tel.: 01 944 3090.*

### Microplate management system

Zymark have introduced a new system package for microplate management which includes all the workstations required to perform a basic microplate assay plus on-bench storage capacity for disposables to allow processing of up to 30 plates per run.

The Microplate Management Software Library, written in EasyLab, is provided with the system and contains subroutines to control workstation activity. An applications program written specifically for the customer's assay is also available. This can be modified so that systematic changes in critical assay variables can be evaluated.

Customers can order from a list of optional workstations to expand the functionality of the system.

The most common application for the Zymark Microplate Management System is ELISAs. Other automated applications include LAL endotoxin testing, drug and pesticide screening, total protein determinations and enzyme quality assurance.

*For further information, contact: Zymark Corporation, Zymark Center, Hopkinton, MA 01748, USA. Tel.: 508 435 9501.*

### Specific protein analyzer from Technicon

Technicon Instruments have announced the launch of the DPA-1, a specific protein analyzer for protein testing in the clinical laboratory which combines the high performance of rate nephelometry and the flexibility of random access analysis. The DPA-1 introduces innovative technology, allowing a multiple number of samples to be monitored whilst simultaneously yielding a throughput of up to 180 reportable patient results per hour.

The fully automated compact system will hold up to 45 patient samples and can be continuously loaded during operation. Each of the 12 resident liquid stable antisera are bar-coded to facilitate automatic entry of lot number, expiry data and calibration parameters. CRP reagent requires no off-line preparation and rheumatoid factor testing is accommodated without any impact on system throughput.

For economy and convenience, the DPA-1 requires only a single point calibration once every month. Specimens are automatically batched so that serum samples are tested separately from cerebrospinal or urine samples, thus ensuring greater productivity. Specific test panels are programmed as desired so that a number of tests are selected by a single keystroke. The entire test menu can be arranged to suit the individual requirements of the user. DPA-1 software incorporates novel algorithms for automatic detection of excess antigen, and samples filling above or below a pre-defined range are also automatically re-run. A bi-directional interface allows transfer of sample programme and patient data between the DPA-1 and an external computer.

*For further information, contact.: Technicon Instruments Company Limited. Tel.: 0256 29181.*

### Biochemical analyser from Technicon

AXON, from Technicon, is a random access, medium throughput biochemistry analyser with high electrolyte capability. The system will analyse 96 samples per hour (576 tests) for ISE and up to four colorimetric channels from an on-board test menu of 60 methods including user-definable assays. Reaction curve monitoring, on-board data storage, full demographic entry and definable report formatting are just four of the features of the user friendly, function drive, full colour software.

Barcode labelling of both reagents and primary sample tubes ensures ease of operation and minimum operator intervention. Approximately 40 methods will be available at launch, including immunoassay and specific proteins. Many of the reagents will require no preparation prior to placement on the system where it will be recognized by its barcode. Likewise barcoded primary containers may be placed in any sequence on the sample tray. Separate positions are provided for STAT and quality control samples.

AXON is designed for economical operation utilizing a combination of low reagent volumes (average 250–300 μl) and an on-board self-laundering system.

*For further information, contact: Technicon, tel.: 0256 29181.*

### Spectroscopic ellipsometer measures film thickness

An instrument for measuring film thickness in the optical and semiconductor industries is now available from Spectrolab. The new instrument, called 'MOSS', is a spectroscopic ellipsometer, capable of measuring the thickness of both single and multiple layers of materials deposited on a variety of substrates. The instrument incorporates a very accurate ellipsometer employing a collimated white light source that is made incident on the samples by way of a rotating polariser. Measurement of elliptically polarised light is made using a fixed analyser polariser and a double monochromator coupled optically to the ellipsometer. The system is therefore able to measure optical parameters at any wavelength between 300 nm and the near infrared, or throughout the infrared when used in conjunction with an FT interferometer.

MOSS is supplied with a complete software package suitable for both on- and off-line analysis, also for simulating the optical parameters of single or multiple films on a variety of substrates. Measurements can be made in real-time using a multichannel analyser enabling film quality and thickness to be measured during the manufacturing process.

*For further information, contact: Spectrolab Limited, P.O. Box 25, Newbury, Berks RG16 8BQ, UK. Tel.: 0635 24808.*

### Servodyne mixer from Cole-Parmer

Cole-Parmer Instrument Company has introduced microprocessor-controlled Servodyne mixers. The Servodyne system maintains and reproduces a constant mixing speed using a precision optical shaft encoder in the mixer head which sends exact motor rotation speed data to the controller. This allows constant speed despite changes in viscosity, temperature and power line voltage. The Servodyne monitors torque and prevents over-stirring or damage to critical fluids by automatically turning off and sounding an alarm. A unique zero torque mode allows monitoring and control of differential torque and the torque reading can be used to determine viscosity directly or, alternatively, a RS-232-C computer link is available to convert the torque output to viscosity units.

The mixer head on the Servodyne system features a through-collet shaft and precision collet for fast setting and infinitely adjustable propeller/shaft height. The controller features a four-digit vacuum fluorescent display that indicates all parameter set points and actual conditions. Set parameters can be stored in the controller's memory as long as line power is maintained.

*For further information, contact: Cole-Parmer Instrument Company, 7425 N. Oak Park Avenue, Chicago, IL 60648, USA. Tel.: 800 323 4340.*

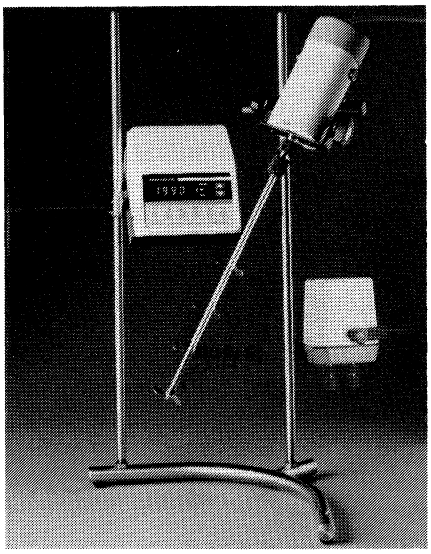

*Servodyne mixer from Cole-Parmer.*

### Automated water analyser reduces labour costs

ChemLab Scientific Products has developed an analyser, particularly for the water industry, which automates four of the most labour-intensive procedures in the analytical laboratory. The CSP Water Analyser simultaneously measures pH, conductivity, colour and turbidity at approximately 60 samples per hour. All the methods employed follow the standard 'Blue Book' procedures, including the colour determination which is carried out on a filtered sample. Up to 80 samples can be loaded on to the turntable at any one time. It is estimated that 2.5 man days per week can be saved by using this novel analyser.

*For further information, contact: ChemLab Scientific Products Ltd, Construction House, Grenfell Avenue, Hornchurch, Essex RM12 4EH, UK. Tel.: 04024 76162.*

### Effluent monitor alarm system

Ionics Ltd have introduced a failsafe alarm unit to draw attention to any failure in carrier gas, compressed air and reagent supplies to its 6800 Series of TC/TOC process analysis monitors. The unit is of particular interest to plants where the monitors are stationed at a remote site or are normally checked infrequently.

The new alarm unit checks sample flow, carrier gas pressures, air flows, furnace temperatures and, for TOC monitors, acid inputs. Mounted in its own enclosure, the alarm system is wired and plumbed to the analyser with a separate terminal strip for contact wiring. The same contact closure (or opening) provides a signal output to the operator's instrument panel. In addition a recorder can be mounted directly into the 6800 Series system to provide a permanent record of signals.

*For further information, contact: Ionics UK Ltd, Carrington Business Park, Carrington, Urmston, Manchester M33 4DD, UK. Tel.: 061 776 4500.*

### Heli-flow combined reaction cell for flow injection analysis

Heli-flow, from WPA, offers a new approach to flow injection analysis, and brings this much discussed analytical technique within the price range of small laboratories.

Designed for reliability and ease of use, Heli-flow gives accurate, reproducible analyses using only a few microlitres of sample and reagent. The spectrophotometric detector, flow cell, two peristaltic pumps, heater and the unique, patented, Heli-flow combined reaction cell, manifold and injection valve are all contained within a single instrument with a footprint of only 350 by 250 mm. The complete Heli-flow system costs less than £3000 compared with more than £10 000 for a conventional flow injection analysis system.

WPA have also introduced two new bench conductivity meters. The CMD630 and CMD650 are both designed for professional applications, with large digital displays and high levels of accuracy, reliability and repeatability. Amongst their features are automatic and manual temperature compensation, multi-range facilities, and variable cell

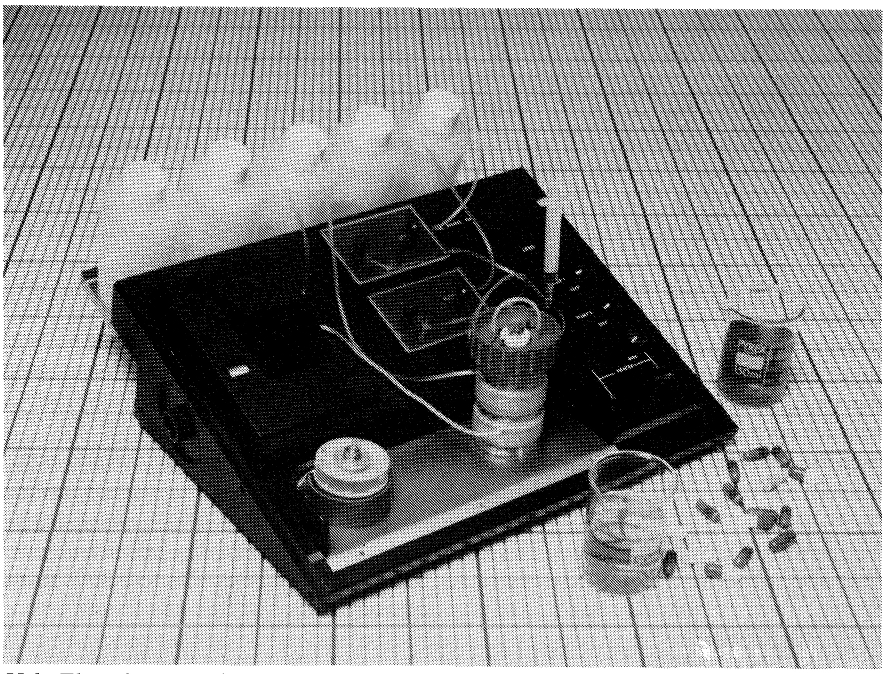

*Heli-Flow from WPA instruments.*

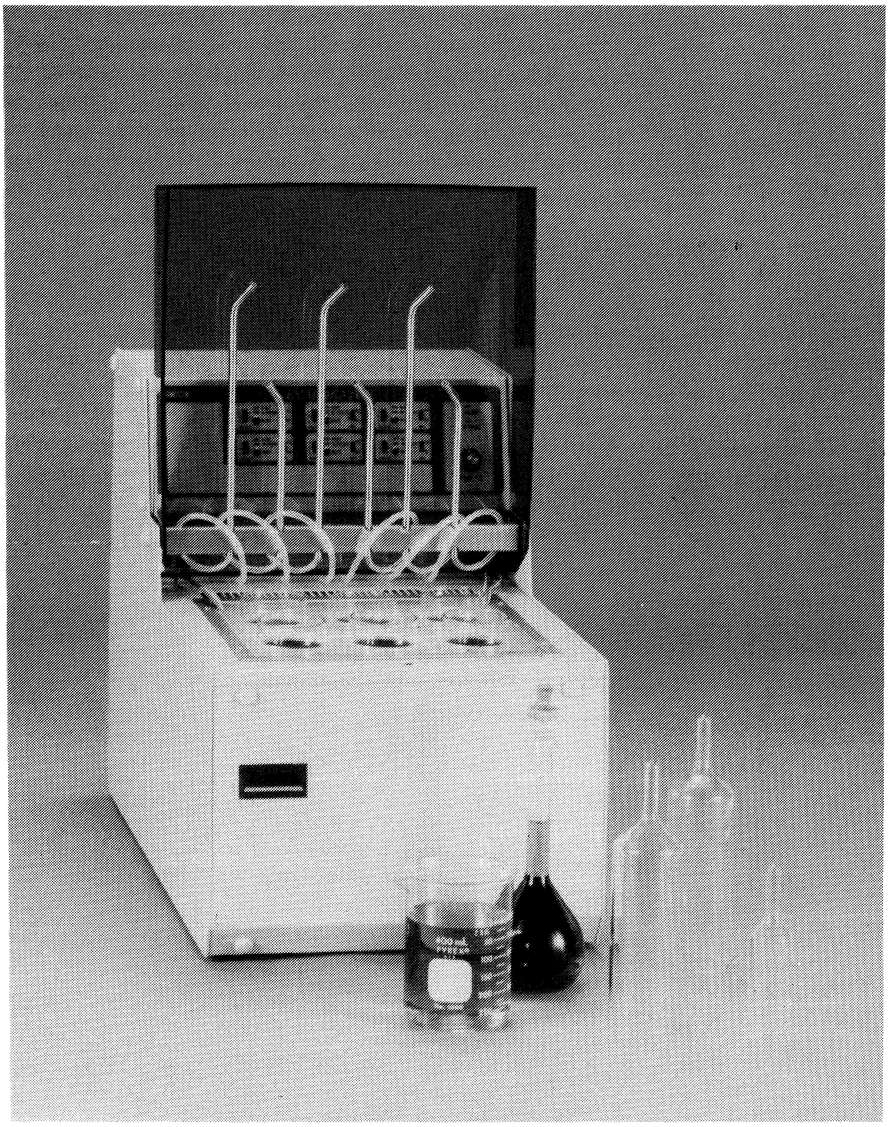

*TurboVap evaporator from Zymark.*

constant and temperature coefficient.

*For further information, contact: WPA Ltd, The Old Station, Linton, Cambridge CB1 6NW, UK. Tel.: 0223 892688.*

### Automatic, high speed endpoint evaporators

Zymark have developed TurboCap automatic, high speed, selectable endpoint evaporators. Key features include: evaporation rates of up to 5 ml $min^{-1}$; selectable endpoint volumes; automatic solvent exchange during evaporation; microprocessor control; multiple sample capacity; laboratory bench operation.

*For further information, contact: Zymark Corporation, Zymark Center, Hopkinton, MA 01748, USA. Tel.: 508 435 9501.*

### Fully automated basket dissolution testing from Zymark

The Zymate II Laboratory Robotics System for automated dissolution testing has been updated to accommodate both the paddle and basket methods. This fully automated dissolution testing system complies to the USP compendial procedures for tablets or capsule oral dosage forms in the pharmaceutical industry. The system combines the Zymate II Robot System and specialized dissolution accessories, the Van-Kel dissolution bath and a UV/VIS spectrophotometer or HPLC of choice.

Communication to the dissolution tester through a unique interface allows the system to set rotation speed for either baskets or paddles. Special telescoping shafts for baskets provide individual starts for each vessel. Internal timers are set for each vessel for accurate and precise timing.

For each assay of basket dissolution testing, the system adds media, runs standards and blanks for UV/VIS, monitors media background, attaches the baskets containing the samples and lowers the shafts, and analyses the samples by UV/VIS or HPLC. The system then washes the vessels and baskets, raises shafts and detaches the baskets, prints the report as it prepares for the next set of assays.

The system is preprogrammed to run the prednisone USP calibrator. Then, easy to follow menu-driven screens allow the input of product specific parameters for individual applications. Product templates have been created to make tasks like running single or multiple point assays, or enteric coated samples that require media change easier.

*For further information, contact: Zymark Corporation, Zymark Center, Hopkinton, MA 01748, USA. Tel.: 508 435 9501.*

### Automated system for the analysis of drugs in biological fluids

Zymark Corporation has announced a robotic system that completely automates the preparation and direct liquid chromatographic injection of biological fluid samples. The system incorporates Zymark's latest improvements in hardware and software including: TurboChip for increased controller processing speed and higher sample throughput; PyTechnology II featuring improved space utilization allowing multi-method operation and new error recovery routines for enhanced reliability; and Tumble Mixer Workstation that reduces emulsion formation when extracting serum, plasma and urine samples. Also included is the Cooled Sample Input Rack to maintain samples at low temperature prior to analysis.

This fully automated system offers improved precision of analytical results since all samples are handled in exactly the same manner and in the same time sequence. PyTechnology's multi-method flexibility lets the operator quickly change the hardware and method software to accommodate more than one procedure on a single benchtop. The drugs in biological fluids system also provides a machine generated audit trail as it verifies all liquid and solid additions via gravimetric tracking.

*For further information contact: Zymark Corporation, Zymark Center, Hopkinton, MA 01748, USA. Tel.: 508 435 9501.*

### New dissolved oxygen meter and oxygen electrode

A dissolved oxygen meter by WPI, model $DO_2M$, features OXEL, a miniature 3 mm (tip diameter) oxygen electrode including a replaceable membrane and electrolyte kit. $DO_2M$ will operate for more than 1000 hours on the same set of AA batteries. The liquid crystal digital panel meter reads directly in percentage oxygen, ppm or nanoamperes of electrode redox current. A recorder output connector allows the user to record the electrode oxygen redox current.

*For further information, contact: World Precision Instruments, 375 Quinnipiac Avenue, New Haven, CT 06513, USA. Tel.: 203 469 8281.*

### On-line analyser measures percent oxygen content

Teledyne's Model 326 provides reliable, economical monitoring of percentage oxygen in a wide range of gases and gas mixtures. Measuring ranges from 0–1% to 0–100% are available with an option of up to 10 atmospheres partial pressure oxygen for hyperbaric applications.

The high accuracy, fast response Model 326R is available in a weather-resistant bulkhead mounting housing or a panel mounting model for general purpose areas. Explosion-proof and intrinsically safe versions can be provided with certain models to CENELEC standards.

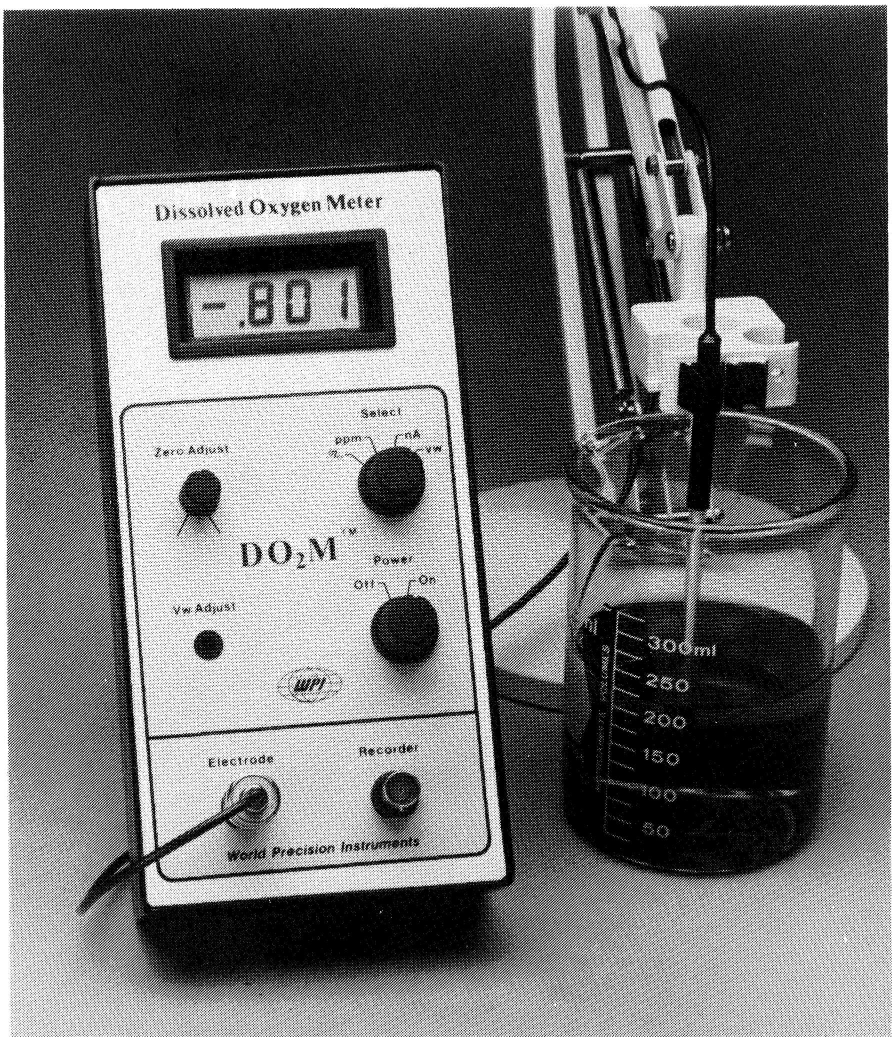

*Dissolved oxygen meter and oxygen electrode from World Precision Instruments.*

The principle of measurement in the Model 326R is the Teledyne micro-fuel cell. This maintenance-free electrochemical device is well proven and is unaffected by almost all gases including hydrocarbons. Added advantages of this sensor are the absolute zero which means no zero gas is required and a linear output which allows air to be used as calibration gas irrespective of measuring range. Further features are its robustness, the low-affect of vibration and changes in flow rate. The standard Model 326 includes three selectable ranges, analog meter readout and O-IVDC signal output, normal options are available.

*For further information, contact: Teledyne Analytical Instruments, The Harlequin Centre, Southall Lane, Southall, Middlesex UB2 5NH, UK. Tel.: 01 571 9596.*

### 9150 Flue gas CO and $O_2$ analysis system

Teledyne Analytical Instruments has dramatically cut the cost of flue gas analysis with the Model 9150, a two-in-one flue gas analyser that monitors both oxygen and carbon monoxide. This dual-sensor analyser is equipped with an air aspirated sample system. It comes standard with three percentage oxygen ranges, 0–5%, 0–10% and 0–25%, and two carbon monoxide ranges, 0–500 ppm and 0–1000 ppm. The system is housed in a rugged enclosure rated NEMA 4, 12, and 13 and IP 55. Options include customised ranges and output signals, and digital or analog meters.

*For further information contact: Teledyne Analytical Instruments, The Harlequin Centre, Southall Lane, Southall, Middlesex UB2 5NH, UK. Tel.: 01 571 9596.*

### New agents for Saftronics 2S

The recent appointment of agents in Norway and Portugal means that Saftronics 2S Ltd of Leeds, manufacturer of solid-state soft starters for AC motors, is now represented in every country throughout Europe and Scandinavia.

The new agents are: (1) Lonne Maskin A/S, Liamyrange 12, N-5090 Nyborg, Norway. Tel.: 010 47 5 186200; and (2) Teclena, AV 25 Abril, Lote 19 R/C Esq, PO Box 249, 2403 Leiria Codex, Portugal. Tel.: 010 351 44 26026/33360.

'We have established this representation in Europe and Scandinavia both because there is a strong demand for our products and in order to provide effective support for our overseas customers,' comments Chris Sanderson, sales and marketing director for Saftronics 2S.

Every agent carries the company's full range of softstarters – the SFTRN SS210 (0·37 to 4·0 kW), SS220 (2.2 to 800 kW) and SS230 (11 to 375 kW) with tacho-feedback for linear acceleration/deceleration. Full training is provided on its products for all agents at the company's Leeds premises.

*For further information, contact: Saftronics 2S Limited, Pearson Street, Leeds LS10 1BQ, UK. Tel.: 0532 457170.*

### Analytical investment by RAPRA

As an extension to the material characterisation capabilities at Rapra, a state of the art infrared spectrometer, fitted with an in-line microscope, has recently been installed.

Infrared spectroscopy is amongst the established analytical methods offered to companies by Rapra and has a key technical input. Visible light microscopy, by virtue of its probing and diagnostic ability, is also an essential technique for material examination. By combining the two techniques in a single instrument Rapra can now provide an unrivalled facility for the identification of contaminants, fibres, defects, etc., and for detailed compositional mapping or morphological studies on polymers.

The microscope component is a full research grade instrument with high performance optics and is fitted with its own dedicated detector. Infrared spectra are recorded through the microscope and can be achieved either by reflection or transmission techniques. A video camera/colour monitor system is employed for sample examination. A photographic record of samples examined can also be obtained.

*For further details of how this can help you, contact: Rapra Technology Limited, Shawbury, Shrewsbury, Shropshire SY4 4NR, UK. Tel.: 0939 250383.*

### P S Analytical move to new larger premises

P S Analytical have recently moved their offices and production facilities to: Arthur House, B4 Chaucer Business Park, Watery Lane, Kemsing, Sevenoaks, Kent TN15 6QY. Originally set up in 1983, P S Analytical has developed a wide range of accessories and modules primarily in the trace elemental analysis market place. These products include the PSA 10.003 Vapour Generator, a range of autosamplers as well as customised automatic analysis systems, for example, automatic density/refractive index systems and automated pH and conductivity systems. End-user sales, as well as OEM accounts, are handled by the manufacturing capabilities.

The improved facilities and additional space will, according to Dr P. B. Stockwell, Managing Director, provide an ideal platform to capitalise on recent developments and to increase production output in line with sales achieved, primarily for mercury determination. The launch of the new Merlin Fluorescence Detector has required increasing production facilities to meet the considerable demand. Since the first units were shipped in October 1988, 20 units have already been installed. The added space will also enable new products already in development to be rapidly brought to the market place.

*For further details of the products in the P S Analytical portfolio and our skills in Laboratory automation, please contact Mr J. E. Bangerter at our new premises. Tel.: 0732 63416; Telex: 957645 PSA G; Fax: 0732 61340.*

